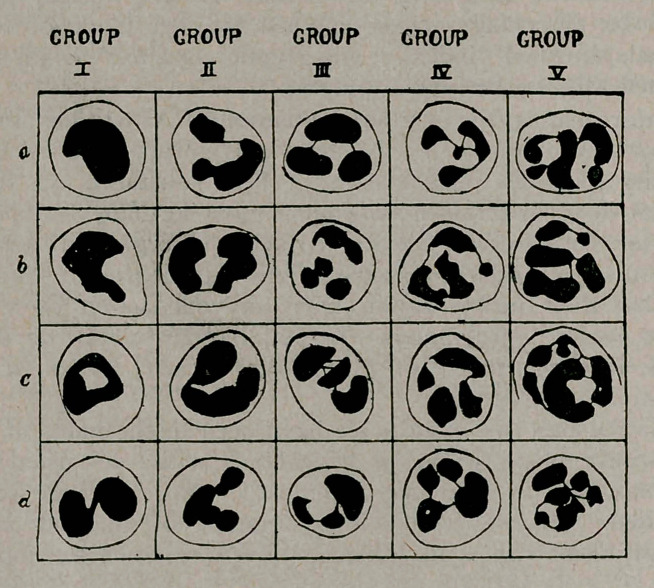# Some Observations on the Arneth Blood Count

**Published:** 1910-11

**Authors:** Charles Gordon Heyd

**Affiliations:** Member of the House Staff of the New York Post-Graduate Hospital


### Some Observations on the Arneth Blood Count

(A preliminary report.)

By CHARLES GORDON HEYD, A.B., M.D.
Member of the House Staff of the New York Post-Graduate Hospital.
(From the Pathological Laboratory.)

(The *Post-Graduate*, September.)

IN the six years following the publication of Arneth's paper an immense amount of work has been done tending to confirm or refute his hypothesis, that the degree of nuclear polymorphism of the polynuclear neutrophilic cells was an accurate index of the resistance of an individual to an infection.

This hypothesis was based upon the common observation that in a smear of pus the leukocytes containing bacteria had, as a general rule, three or more nuclei. In other words, phagocytosis reached its best development in those cells which showed marked polynucleosis, and was directly proportional to the number of "nuclei" in the leukocytes. A cell with three nuclei or three distinct nuclear masses would be a more active and energetic phagocyte than the cell with only two nuclei; the one with four more than the one with three, etc. Upon such a supposition one could determine the degree of nuclear polymorphism in terms of per cent. by dividing a count of one hundred polymorphonuclear leukocytes into five groups depending upon whether the nucleus showed one, two, three, four, or five nuclear masses. From the percentages thus obtained the resistance of the individual, so-called "index of phagocytic capacity," could be determined as a mathematical quantity.[1]

Unfortunately, the original contention of Arneth has not been by any means proved. Nuclear polymorphism does not of necessity parallel phagocytosis. The resistance of an individual to an infection is dependent upon so many factors, such as variety of infectious agents; virulence of organism; acuteness of the process, and the relative or absolute degree of immunity, etc., that their interpretation upon any one aspect of a blood picture seems unwarranted. Judged by our work, we do not believe that the index of phagocytic capacity is an accurate estimation of resistance.

For the past six months we have been in the habit of doing an Arneth blood count in connection with our usual blood examinations. In following a number of surgical cases we were impressed with the common association of marked nuclear polymorphism with acute purulent infections, such as empyema,

1. For example: Normal counts by Arneth as quoted by Chace (*Post-Graduate*, Vol. 22, p. 509).

| | Leukocytes | Group | | | | | Index of phagocytic capacity |
|---|---|---|---|---|---|---|---|
| | | I | II | III | IV | V | |
| 1 | 5300 | 4% | 21% | 48% | 23% | 4% | 2782 |
| 2 | 5400 | 7% | 39% | 36% | 16% | 2% | 2051 |
| 3 | 5000 | 9% | 47% | 37% | 9% | 0% | 1510 |
| 4 | 5000 | 9% | 45% | 38% | 8% | 0% | 1510 |
| 5 | 10000 | 9% | 46% | 32% | 13% | 0% | 3150 |

In determining the index of phagocytic capacity we assumed that 70 per cent. is the normal number of polynuclear leukocytes in normal blood. Therefore 70 per cent. of 5300 equals 3710.

And of these 3710 cells 75 per cent. (summation of groups, three, four, and five) are active phagocytes:

75 per cent. of 3710 equals 2782, which represents the index of phagocytic capacity.

appendicitis, osteomyelitis, cholecystitis, etc. As a result, the following questions came up: could we diagnosticate the presence of pus by means of the absolute or relative increase of the cells of the fourth and more especially the fifth groups? Was there any parallelism between a marked polynucleosis and a high differential count of polymorphonuclear leukocytes? Was there any association with the iodophilic or glycogenic reaction of the white blood cells?

In making the count it was necessary to establish some arbitrary standard of nuclear division, in order to place a definite and uniform value to varying counts from day to day. Arneth seems to have counted each nuclear lobe as a nucleus while Knowatzki counted as distinct nuclei those nuclear masses separated by a clear space. We determined to count as separate nuclei only such nuclear masses as were absolutely detached from their neighbors or joined by barely perceptible chromatic fibrillæ. With such a clear-cut standard it is surprising how few cells fall within the fourth group and especially seldom in the fifth group in a normal blood count. In our series over eighty Arneth counts were made, many upon patients having a normal blood count and were taken as part of the routine examination of every patient admitted to the medical, and to a less extent, the surgical wards. Eliminating the normal as devoid of interest we have thirty-four counts upon twenty-three different patients, as follows:

| No. | Leuko-cytes | Poly-nuclears | Group I | II | III | IV | V | Remarks |
|---|---|---|---|---|---|---|---|---|
| | | Per Cent | Per cent | Per cent | Per cent | Per cent | Per cent | |
| 1 | 7200 | 85 | 26 | 38 | 23 | 11 | 2 | Pronounced secondary anemia, dysentery.—mucopurulent stools. |
| 2 | 8000 | 86 | 38 | 40 | 15 | 7 | 0 | Child aet. 6. Nephritis, hemorrhagic, no etiological factor could be determined. Recovery. |
| 3 | 3600 | 74 | 81 | 15 | 4 | 0 | 0 | Typhoid, no complications. Recovery. |
| 4 | 16000 | 91 | 47 | 42 | 10 | 1 | 0 | Gastric hemorrhage—ulcer, question of carcinoma? |
| 5 | 6700 | 87 | 18 | 42 | 35 | 5 | 0 | Diabetes—cataract, coma, death, |
| 6 | 25380 | 90 | 53 | 38 | 8 | 1 | 0 | Lobar pneumonia. |
| | 25994 | 94 | 38 | 36 | 19 | 5 | 2 | 2nd day. |
| | 19270 | 94 | 67 | 27 | 4 | 2 | 0 | 5th day—crisis—recovery. |
| 9 | 21280 | 82 | 63 | 29 | 6 | 2 | 0 | Lobar pneumonia, 12th day, delayed resolution. |
| | 9300 | 76 | 32 | 39 | 27 | 2 | 0 | 15th day, resolution. Recovery. |
| 11 | 20000 | 88 | 45 | 33 | 17 | 3 | 2 | Lobar pneumonia, 2nd day, recovery. |
| 12 | 20000 | 91 | 50 | 29 | 14 | 5 | 2 | Lobar pneumonia, 2nd day, recovery. |
| 13 | 16000 | 80 | 32 | 33 | 23 | 8 | 4 | Lobar pneumonia, 2nd day, recovery. |
| 14 | 25000 | 90 | 77 | 20 | 3 | 0 | 0 | Child aet. 4 years, 4 months, entered the hospital in comatose state. 18 hours after admission both lungs solidified. Death in 36 hours. |
| 15 | 14600 | 74 | 40 | 32 | 24 | 4 | 0 | Senile gangrene. operation, death from shock within 20 minutes. |
| 16 | 9300 | 65 | 17 | 36 | 36 | 11 | 0 | Stricture of esophagus. Diagnosis undetermined—lues or carcinoma? |
| 17 | 18600 | 89 | 44 | 35 | 19 | 2 | 0 | Unresolved pneumonia, question of empyema, decided as unresolved pneumonia, recovery. |
| 18 | 36000 | 94 | 41 | 41 | 12 | 3 | 1 | Tubercular kidney — septicemia — mixed infection. Death in 36 hours. No autopsy. |
| 19 | 15300 | 86 | 57 | 39 | 4 | 0 | 0 | Tubercular kidney, oper. abscess with extensive destruction of renal parenchyma, large amount of pus. Recovery. |
| 20 | 20000 | 40 | 20 | 50 | 25 | 5 | 0 | Child aet. 18 months. This case with extreme anemia and marked blood polymorphism suggested Von Jaksch's disease—anemia infantum pseudo-leukemica. Came in with a frontal abscess, late L. fibrinous pleurisy. Tapped, recovery. Strong probability of tuberculosis. |
| 21 | 16000 | 90 | 49 | 25 | 18 | 5 | 0 | Came in as appendicitis; rash next morning showed scarlatina. |
| 22 | 15000 | 94 | 38 | 35 | 17 | 7 | 3 | 12 hours after radical mastoid operation. Free discharge, recovery. |
| 23 | 14000 | 85 | 41 | 41 | 18 | 0 | 0 | Admitted. |
| | 14000 | 91 | 47 | 31 | 17 | 3 | 2 | |
| | 13500 | 88 | 48 | 43 | 9 | 0 | 0 | Discharged. Endometritis—puerperal sepsis, after six weeks, recovery. |
| 26 | 18000 | 75 | 73 | 27 | 0 | 0 | 0 | Pyosalpinx, six weeks' duration. Marked adhesions. Rupture during removal. Recovery. |
| 27 | 22000 | 74 | 58 | 28 | 8 | 4 | 2 | Child aet. 18 months. Intussusception—ileocecal, 18 hours duration; bowel compromised but not gangrenous. Recovery. |
| 28 | 28000 | 86 | 33 | 46 | 21 | 0 | 0 | Appendicitis — chronic — six weeks' duration. Clinically only pain and tenderness. Oper. appendical abscess, well walled-off, appendix not sought. Recovery. |
| 29 | 23600 | 93 | 42 | 29 | 24 | 5 | 0 | Appendicitis—acute, beginning gangrene and perforation. No free pus. Recovery. |
| 30 | 13000 | 90 | 30 | 45 | 22 | 3 | 0 | Appendicitis—acute. No gangrene, no pus. Recovery. |

| No. | Leuko-cytes | Poly-nuclears | Group I | Group II | Group III | Group IV | Group V | Remarks |
|---|---|---|---|---|---|---|---|---|
| | | Per Cent | Per cent | Per cent | Per cent | Per cent | Per cent | |
| 31 | 12000 | 82 | 33 | 39 | 20 | 8 | 0 | Appendicitis—acute, beginning gangrene and perforation, adhesions, no pus, recovery. |
| 32 | 23000 | 83 | 31 | 26 | 32 | 7 | 4 | Appendicitis—acute, 16 cc. of pus. Recovery. |
| 33 | 20000 | 87 | 34 | 40 | 15 | 9 | 2 | Appendicitis—acute, gangrene and empyema. Some free pus. Recovery. |
| 34 | 14000 | 80 | 53 | 21 | 14 | 2 | 0 | Cholecystitis *vs.* appendicitis, with hysteria. Small per cent. in group four and five against the latter. No operation. Recovery in four days. |

Five of the cases are in children, *viz.*, 2, 14, 20, 21, 27.

Eleven of the thirty-four counts show from one to four per cent. in group five. Of the eleven one was a pronounced form of secondary anemia—secondary to some undetermined intestinal lesion; four were from cases of pneumonia; one from intussusception and five from pus conditions, mostly appendicitis and characterised by the presence of "free pus." Only two pus conditions Nos. 26 and 28 did not have any representation in group five. The lack of group five cells in these two cases is probably due to a variety of factors among which are: variety of infecting organism; virulence of organism; chronicity of the process; presence of marked adhesions, thereby preventing septic absorption. It would seem that the presence of group five cells was dependent upon the acuteness of any given process and that, in general, an acute pyogenic infection would show some cells in the fifth group. If a count of one hundred cells showed some in group four with from two to six per cent. in group five, we were in the habit of considering it an indication of a pus focus. It is interesting to note that, as a rule, with acute pyogenic infections there was a tendency for diminution of percentage in the first two groups, while groups three, four and five showed correspondingly increased percentages.

In pneumonia, there is a striking uniformity of high leukocytic count, high differential count and a small percentage of cells in group five. This is especially true on the second and third day, and with the crisis the group five reaction disappears synchronously with the drop in leukocytes and differential count. All our pneumonia cases with group five cells recovered and the one fatal case occurred where there were no cells in the fifth group. The presence of some cells in the fifth group evidently evidenced a good resistance on the part of the individual.

Ten cases without pus—pneumonia excepted—showed no cells in group five, *viz.*, 2, 3, 4, 5, 15, 16, 21, 29, 30, 34. Case 27, that of intussusception, showed group five cells but no pus. This case marks an exception to the rule. Of the two tubercular kidney cases, one showed only one per cent. in group five while the other was group five negative, although both presented evidence of mixed infections.

In general, there is some parallelism between a high polynuclear differential and the presence of cells in group five, although there are cases with a high differential count which are devoid of any cells in the fifth group. So far as the glycogen reaction is concerned, our observations are too fragmentary to admit of any attempt at classification. A hasty survey of a few cases leads us to believe that there exists a moderate parallelism between a good idophilic reaction and the presence of a positive group five. In acute infections characterized by free pus we generally had from one to five per cent. of cells in group five. The presence of these group five cells seems to be dependent upon a peculiar stimulus which is lacking in chronic pus collections and in pus of gonorrheal origin.

### CONCLUSIONS.

1. The Arneth count as an index of resistance is untenable.
2. In acute infections, pneumonia excepted, the presence of cells in the fifth group gives us valuable information as to the probability of there being a pus collection. In about two-thirds of acute appendicitis the degree of nuclear polymorphism is directly proportional to the acuteness and virulence of the process.
3. In pneumonia the blood picture conforms to that existing in acute purulent infections.
4. In chronic infections the Arneth count admits of no safe deductions.

---

Practical Points in the Management of Poliomyelitis and its Sequelae.—Henry Ling Taylor, New York, thinks that time is wasted in treating anterior poliomyelitis with massage and electricity. These agents have little effect. The prevention of deformities and their correction is of the utmost importance. But rest in bed should be maintained for a long time in order to prevent stretching of the affected muscles by the weight of the paralysed limbs. Many cases of scoliosis in young adults have been traced to an early attack of poliomyelitis. The abdominal muscles are often affected and contribute to the scoliosis. As soon as the acute symptoms have subsided measures should be undertaken not to allow of stretching of the paralysed muscles by the weight of the limbs. Support should be fitted. After deformity has taken place it is important to correct it by apparatus and operations. There is no advantage in exercising the opposing muscles, but quite the reverse. Improvement may be obtained even in cases of long standing by these measures, and a fair amount of motion obtained by careful balancing of the muscles.—*Medical Record*, October 15, 1910.

## Figures and Tables

**Figure f1:**